# Blood-based DNA methylation and exposure risk scores predict PTSD with high accuracy in military and civilian cohorts

**DOI:** 10.21203/rs.3.rs-3952163/v1

**Published:** 2024-02-15

**Authors:** Agaz Wani, Seyma Katrinli, Xiang Zhao, Nikolaos Daskalakis, Anthony Zannas, Allison Aiello, Dewleen Baker, Marco Boks, Leslie Brick, Chia-Yen Chen, Shareefa Dalvie, Catherine Fortier, Elbert Geuze, Jasmeet Hayes, Ronald Kessler, Anthony King, Nastassja Koen, Israel Liberzon, Adriana Lori, Jurjen Luykx, Adam Maihofer, William Milberg, Mark Miller, Mary Mufford, Nicole Nugent, Sheila Rauch, Kerry Ressler, Victoria Risbrough, Bart Rutten, Dan Stein, Murrary Stein, Robert Ursano, Mieke Verfaellie, Erin Ware, Derek Wildman, Erika Wolf, Caroline Nievergelt, Mark Logue, Alicia Smith, Monica Uddin, Eric Vermetten, Christiaan Vinkers

**Affiliations:** University of South Florida College of Public Health, Genomics Program; Emory University Department of Gynecology and Obstetrics; Boston University School of Public Health; Broad Institute of MIT and Harvard, Stanley Center for Psychiatric Research; University of North Carolina at Chapel Hill, Carolina Stress Initiative; Robert N Butler Columbia Aging Center, Columbia University; University of California San Diego, Department of Psychiatry; Brain Center University Medical Center Utrecht, Department of Psychiatry; Alpert University, Brown University; Biogen Inc., Translational Sciences; University of Cape Town, Department of Pathology; Harvard Medical School, Department of Psychiatry; Netherlands Ministry of Defence, Brain Research and Innovation Centre; The Ohio State University, Department of Psychology; Harvard Medical School, Department of Health Care Policy; The Ohio State University, College of Medicine, Institute for Behavioral Medicine Research; University of Cape Town, Department of Psychiatry & Mental Health; Texas A&M University College of Medicine, Department of Psychiatry and Behavioral Sciences; Emory University, Department of Psychiatry and Behavioral Sciences; UMC Utrecht Brain Center Rudolf Magnus, Department of Psychiatry; University of California, San Diego; VA Boston Healthcare System, TRACTS/GRECC; Boston University School of Medicine, Psychiatry; University of Cape Town, Neuroscience Institute; Alpert Brown Medical School, Department of Emergency Medicine; Emory University, Department of Psychiatry & Behavioral Sciences; Harvard Medical School, Department of Psychiatry; University of California San Diego, Department of Psychiatry; Maastricht Universitair Medisch Centrum, School for Mental Health and Neuroscience, Department of Psychiatry and Neuropsychology; University of Cape Town, Department of Psychiatry & Mental Health; University of California San Diego, Department of Psychiatry; Uniformed Services University, Department of Psychiatry; Boston University School of Medicine, Psychiatry; University of Michigan, Population Studies Center; University of South Florida College of Public Health, Genomics Program; VA Boston Healthcare System, National Center for PTSD; University of California San Diego, Department of Psychiatry; Boston University School of Public Health; Emory University Department of Gynecology and Obstetrics; University of South Florida College of Public Health, Genomics Program; Leiden University Medical Center, Department of Psychiatry; Amsterdam Neuroscience, Mood, Anxiety, Psychosis, Sleep & Stress Program

**Keywords:** DNA methylation, Machine learning, PTSD, Risk scores

## Abstract

**Background:**

Incorporating genomic data into risk prediction has become an increasingly useful approach for rapid identification of individuals most at risk for complex disorders such as PTSD. Our goal was to develop and validate Methylation Risk Scores (MRS) using machine learning to distinguish individuals who have PTSD from those who do not.

**Methods:**

Elastic Net was used to develop three risk score models using a discovery dataset (n = 1226; 314 cases, 912 controls) comprised of 5 diverse cohorts with available blood-derived DNA methylation (DNAm) measured on the Illumina Epic BeadChip. The first risk score, exposure and methylation risk score (eMRS) used cumulative and childhood trauma exposure and DNAm variables; the second, methylation-only risk score (MoRS) was based solely on DNAm data; the third, methylation-only risk scores with adjusted exposure variables (MoRSAE) utilized DNAm data adjusted for the two exposure variables. The potential of these risk scores to predict future PTSD based on pre-deployment data was also assessed. External validation of risk scores was conducted in four independent cohorts.

**Results:**

The eMRS model showed the highest accuracy (92%), precision (91%), recall (87%), and f1-score (89%) in classifying PTSD using 3730 features. While still highly accurate, the MoRS (accuracy = 89%) using 3728 features and MoRSAE (accuracy = 84%) using 4150 features showed a decline in classification power. eMRS significantly predicted PTSD in one of the four independent cohorts, the BEAR cohort (beta = 0.6839, p-0.003), but not in the remaining three cohorts. Pre-deployment risk scores from all models (eMRS, beta = 1.92; MoRS, beta = 1.99 and MoRSAE, beta = 1.77) displayed a significant (p < 0.001) predictive power for post-deployment PTSD.

**Conclusion:**

Results, especially those from the eMRS, reinforce earlier findings that methylation and trauma are interconnected and can be leveraged to increase the correct classification of those with vs. without PTSD. Moreover, our models can potentially be a valuable tool in predicting the future risk of developing PTSD. As more data become available, including additional molecular, environmental, and psychosocial factors in these scores may enhance their accuracy in predicting the condition and, relatedly, improve their performance in independent cohorts.

## Background

Posttraumatic stress disorder (PTSD) is a psychiatric disorder that can develop after experiencing or witnessing a life-threatening event such as a war/combat, natural disaster, violence, or serious accident. PTSD occurs in 5–10% of the population, and females are twice as likely to experience PTSD as males [1]. PTSD commonly occurs together with other psychiatric disorders [2, [3, [4, [5] and has also been associated with other health conditions such as accelerated aging [6, [7], cardiovascular and metabolic disorders [8, [9], and poor physical health [10]. Consequently, the individual and societal burden caused by PTSD is quite high [11, [12]. Identifying individuals at elevated risk of PTSD would enhance the ability to develop timely preventive strategies and therapies for this disorder, which would, in turn, help to reduce the associated disease burden.

Incorporating genomic data into risk prediction has become an increasingly useful approach for rapid identification of individuals most at risk for complex disorders such as PTSD. In particular, polygenic risk scores (PRS) have been evaluated in both research and clinical contexts to estimate risk to develop complex disorders, including coronary artery disease, breast cancer, Type 2 diabetes, and Alzheimer’s Disease (reviewed in [13]). These genetically-based risk scores are attractive as they access lifetime risk for a particular disorder and leverage variation across hundreds to thousands of variants. However, PRSs typically explain only a small proportion of variance in risk for a particular disorder and do not capture environmental factors that influence risk or detect the effect of disease progression itself [14], both of which may be important to identifying individuals at highest risk for disease.

In contrast, risk scores based on DNA methylation levels, which are modifiable and dynamic, can potentially convey more information about disease risk. A growing literature has shown that approaches originally developed for generating PRS can be adapted for DNA methylation data (reviewed in [15, [16]). The resulting methylation risk scores (MRS) have been shown in some cases to be more indicative of current disease state [17] and health-related phenotypes [18], as well as more predictive of future disease risk [19], than PRS-based approaches. Indeed, for PTSD, which requires an environmental exposure—trauma/shocking event —to meet the requirements for a diagnosis, MRS-based risk scores that capture the differential effects of this exposure may be particularly informative for identifying trauma-exposed individuals most at risk for the disorder.

To this end, here we leverage a large, ancestrally diverse set of cohorts to take a first step toward developing methylation risk scores for PTSD. We focus specifically on developing scores that distinguish between those with vs. without the disorder (i.e., a diagnostic MRS that correctly classifies current cases vs. trauma-exposed controls), and attempt to replicate these MRS in multiple external validation cohorts. We further test whether these diagnostic risk scores have prognostic value, i.e., can predict future PTSD among individuals prior to trauma exposure. Finally, to gain insight into potential mechanisms, we investigate the biological significance associated with the CpGs that comprise the MRS.

## Methods

### Cohorts

In order to maximize the available data from which to develop risk scores using machine learning approaches, we created a discovery cohort comprised of 1226 individuals drawn from five cohorts (Table 1). Two of these cohorts are civilian— Detroit Neighborhood Health *Study (DNHS)* and *Grady Trauma Project (GTP)*, and *three cohorts are military* — Army Study to Assess Risk and Resilience in Servicemembers (Army STARRS), Marine Resilience Study (MRS I&II), and Prospective Research in Stress-related Military Operations (PRISMO). Details about each cohort are given in the supplementary file. The overall workflow of the pre-processing and methods combining data from the five cohorts is shown in supplementary file (Figure S1).

### Quality Control (QC) Procedures

DNAm from whole blood was measured using the Illumina MethylationEPIC BeadChip following the manufacturer’s recommended protocol. Raw DNAm β values were obtained, and a sex check was conducted using the *minfi* R package [20] to eliminate any sex-discordant samples. Quality control (QC) was performed on each cohort separately, using a standardized pipeline as previously described [21], producing 818,691 probes that passed QC. Normalization was carried out using the single-sample Noob (ssNoob) method in the *minfi* R package [20]. Furthermore, *ComBat* adjustment was performed, using an empirical Bayesian framework implemented in the *SVA* R package [22, [23] to reduce the likelihood of bias due to known batch effects (chip and position), while preserving the variation for age, sex (if applicable), and PTSD. The resulting QC’d data was used in subsequent analyses.

### Estimation of Covariates

#### Smokingscores.

Studies have linked methylation at many genomic loci to smoking status [24, [25, [26, [27, [28, [29]. Therefore, to adjust for DNA methylation differences related to smoking, we calculated smoking scores from DNA methylation data based on the weights obtained from 39 CpGs located at 27 loci, as previously described [30].

#### Cell proportions.

It is important to consider cellular heterogeneity in EWAS [31] since whole blood contains various cell types, each with its own DNA methylation profile [32, [33]. To address this, cell proportions (CD4+T, CD8+T, Natural Killer (NK), B-cells, monocytes, and neutrophils) were estimated using reference data [34] and the Robust Partial Correlation (RPC) method implemented in the *EpiDISH* R package [35].

#### Ancestry principal components.

Several DNA methylation studies have found variations in DNA methylation levels among different populations (race/ethnicity) at certain CpG sites [36, [37, [38, [39, [40, [41]. Therefore, to account for population stratification in DNA methylation studies, ancestry principal components (PCs) were generated from methylation data using a subset of CpGs in close proximity to SNPs in data from the 1000 Genomes Project [42, [43]. As previously reported [42, [43], PC 2 and 3 were the components most correlated with ancestry and thus, used to adjust for population stratification in this study.

#### Covariate adjustment

To ensure accuracy of models, missing values in DNAm were imputed using the mean method [44, [45]. We then adjusted the DNAm data for potential confounding factors, including cell composition, ancestry, smoking score, sex (if applicable), and age, for models 1 and 2 (described below). The adjustment was made for each CpG by regressing out the covariates using linear regression and then replacing the values of CpG with the corresponding residuals [46]. For model 3 (described below), we also accounted for the two exposure variables of interest, cumulative trauma and childhood trauma, in addition to the covariates used in models 1 and 2. This was done to account for any differences related to exposure variables in individual cohorts.

### Analysis

#### Overall Approach.

Our goal was to develop a series of models based on important (i.e. set of features with best classification accuracy) methylation- and (in some cases) exposure-related features to classify PTSD that would then be used to derive risk scores with which to predict PTSD. To train the models, we utilized unique, trauma exposed participants from the discovery cohort in a cross-sectional approach. Model 1 was designed to classify PTSD by including two exposure variables—cumulative trauma (number of traumatic events experienced) and childhood trauma (experienced at < 18 years of age)—along with DNAm data, as increasing levels of exposures are known to substantially increase the risk of developing PTSD [47, [48, [49, [50] and were thus hypothesized to contribute high predictive power to our model. The purpose of Model 2 was to classify PTSD using only DNAm data, without relying on the discriminatory power of cumulative trauma or childhood trauma; this model would enable potential application to cohorts in which only DNAm data were available. Model 3 was developed with a unique purpose, distinct from Model 1. Namely, it was created to account for variations in exposure variables among individual cohorts. In this model, exposure variables were intentionally excluded from the analysis because they were used as covariates in DNAm data adjustment. While Model 3 addresses the challenge of cohort-specific variations, it does not possess the same predictive power as Model 1, which incorporates these exposure variables. The adjusted data was then subjected to the following analysis processes.

### Feature Selection and Scaling

We used *SelectKBest* in *Scikit-learn* [51], a univariate feature selection approach. This method computes ANOVA F-values based on univariate statistical tests to identify the best features in relation to a particular phenotype. We identified the most important features from DNAm and exposure variables (in cases of Models 1 & 2) based on the highest score. For Model 3, we selected features solely from DNAm data.The feature selection process was repeated 500 times, ranging from 10 to 5000 features with a 10-feature increment each time to determine the optimal feature set for the Elastic Net model best accuracy. As different studies/cohorts used different instruments to measure cumulative trauma and childhood trauma, we normalized the data using a min-max scale that ranged from [0, 1].

### Training and Testing

In order to identify the best model to classify PTSD and determine risk scores, we trained three popular machine learning models —Random Forest, Lasso, and Elastic Net on 75% of the data, and then tested them on the remaining 25% using the *Scikit-learn* [51] framework. We also conducted a 10-fold cross-validation on training and testing data to evaluate the effectiveness of the models (Figure S1.1). After selecting the most accurate model (Model 1), we used important features (methylation and exposure variables) identified during the feature selection process to classify PTSD. Following covariate adjustment, we re-ran the feature selection process to identify important features for Model 3. Performance of the models was assessed using accuracy, precision, recall, f1-score and area under the curve (AUC) metrics.

### Risk scores

Risk scores, which are the weighted sum of the important features, were created on discovery cohort test data (25%), using feature weights (effect sizes) from training data (75%) to test for an association between risk scores and PTSD. Model 1 contributed to the development of exposure and methylation risk scores (eMRS), whereas Model 2 provided methylation-only risk scores (MoRS). Finally, Model 3 led to the creation of methylation-only risk scores with adjusted exposure variables (MoRSAE).

A logistic model was employed to test for an association between risk scores (eMRS, MoRS and MoRSAE) and PTSD, and the Nagelkerke approach was used to assess the models’ resulting R-Squared (R^2^) values. Further, in cohorts with available pre-deployment DNA methylation, a logistic model was used to predict post-deployment PTSD using risk scores calculated from *pre-deployment* DNAm data and exposure data (Army STARRS, MRS I&II; note that these participants had their post-deployment DNAm data included in the discovery cohort analyses described above). For all analyses, a Wilcox rank-sum test was used to assess differences in risk scores between cases and controls. To investigate correlation among study variables in discovery and independent cohorts, Pearson’s and point-biserial correlation was used, as appropriate.

### Independent Validation

To validate the risk scores, we tested their ability to distinguish those with vs. without PTSD in four independent, external cohorts using the same pre-processing and covariate adjustment pipeline as in the discovery cohort. Brief descriptions of the external cohorts (NCPTSD-TRACTS, BEAR, DCHS and PROGrESS) are provided in the Supplementary file. We utilized weights from significant features identified in models 1, 2, and 3 of the discovery cohort to generate risk scores i.e., eMRS, MoRS, and MoRSAE in the external cohorts. Similar to the discovery cohorts, we conducted Pearson and Point-Biserial correlation tests, association tests using logistic regression model, and Wilcox rank-sum tests on external cohorts.

### Enrichment Analysis

To investigate the biological significance of the important CpGs identified in the feature selection step, we performed Gene Ontology (GO) enrichment analysis and Kyoto Encyclopedia of Genes and Genomes (KEGG) pathway analysis using missMethyl [52]. Gene ontologies and KEGG pathways that reached a nominal significance level of p < 0.05 were considered important.

## Results

### Description of Discovery Cohort

Table 1 provides a summary of the demographic characteristics and clinical information of all participants (n=1226) in the discovery cohort with current PTSD. More information about cumulative and childhood trauma is provided in Table S1. A slight majority of participants were male (n=629). Two cohorts, DNHS and GTP, were comprised mostly of African Americans, while the remaining three cohorts were predominantly of European ancestry. In all cohorts, a significant difference in PTSD symptom severity was observed between cases and controls (p<0.05). With the exception of Army STARRS, childhood trauma also demonstrated a significant difference between PTSD cases and controls (p<0.05) in all cohorts. Finally, a significant difference was observed in cumulative trauma between cases and controls in DNHS and GTP (p < 0.001).

### Development of Methylation Risk Scores to distinguish those with vs. without PTSD

**Figure 1:**
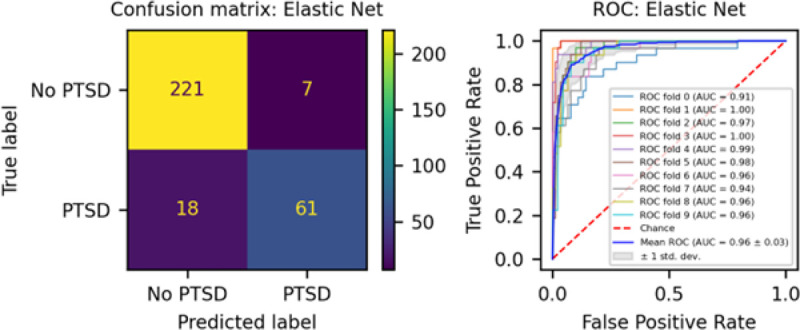
The confusion matrix for Model 1 displays an accuracy of 92% on test data (N = 307), while the ROC curve indicates an AUC of 96% during the 10-fold cross-validation using all data (N = 1226).

**Figure 2:**
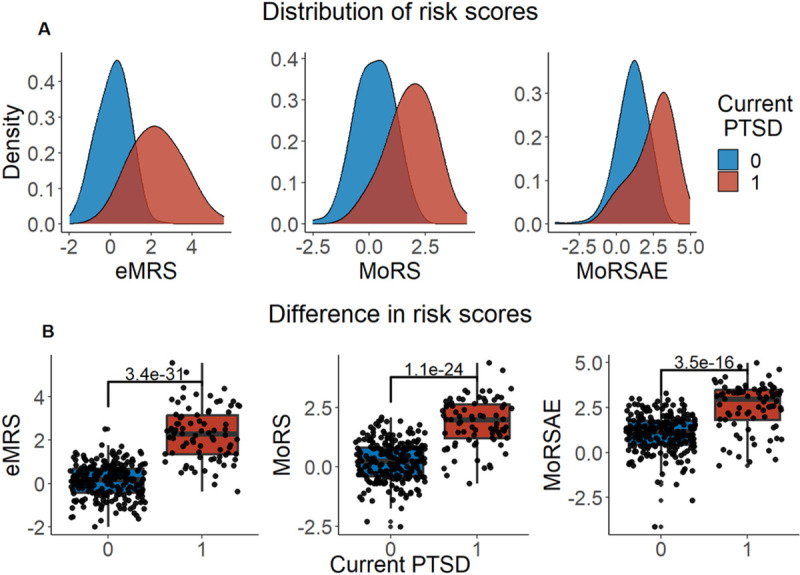
Distribution and variation of risk scores between cases and controls in test data (N =307) in figure legend, 0 is No PTSD and 1 is PTSD. A) The distribution of risk scores for Models 1, 2, and 3 is shown for both cases and controls. B) The difference in risk scores between cases and controls is displayed. Model 1 calculates exposure and methylation risk scores (eMRS), while Model 2 calculates risk scores based only on methylation variables (MoRS). Model 3 calculates risk scores based on methylation variables adjusted for exposure variables (MoRSAE). The risk scores are higher in PTSD cases compared to controls. The Wilcox test confirms a significant difference in risk scores between cases and controls with p < 0.001 for all models (1, 2, and 3).

**Figure 3:**
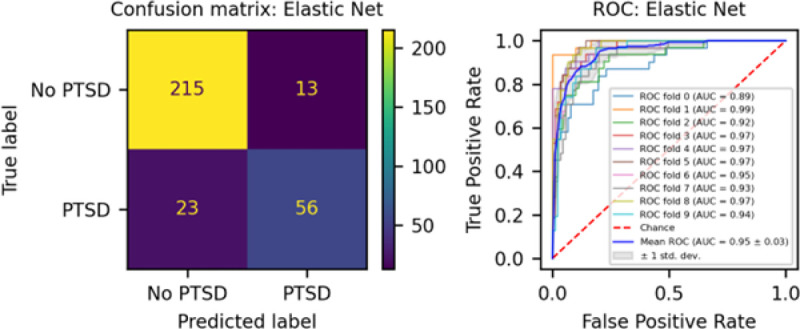
The confusion matrix for Model 2 displays an accuracy of 89% on test data (N = 307), while the ROC curve indicates an AUC of 95% during the 10-fold cross-validation using all data (N = 1226).

**Figure 4:**
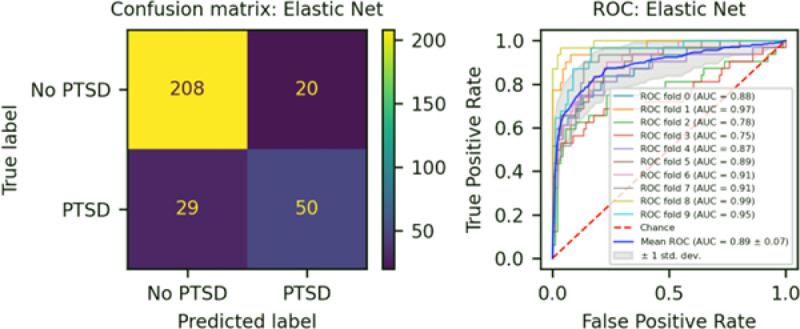
The confusion matrix for Model 3 displays an accuracy of 84% on test data (N = 307), while the ROC curve exhibits an AUC of 89% during the 10-fold cross-validation process using all data (N = 1226).

We developed three different risk scores with the goal of distinguishing those with vs. without PTSD using machine learning approaches. Our first model, eMRS, included both exposure and DNA methylation variables and identified 3730 features (3728 CpGs, cumulative trauma, and childhood trauma) as important in the discovery cohort. Using these 3730 features, Elastic Net approaches were employed to achieve the best accuracy (92%; Figure 1), precision (91%), recall (87%), and f1-score (89%); Table 2 (See Fig. S2 for AUCs with Lasso and Random Forest approaches). The eMRS significantly predicted PTSD (beta = 2.64, p < 0.001), R^2^ =0 .70), with higher eMRS values in PTSD cases than controls (p<0.001; Figure 2A, left plot). Our second MoRS model, based solely on the 3728 methylation features in model 1, accurately classified PTSD with 89% accuracy and had an AUC of 95% (Figure 3; Table 2). Additionally, the precision, recall, and f1-score were at 86%, 83%, and 84%, respectively, as shown in Table 2. As with eMRS, the MoRS significantly predicted PTSD (beta =2, p < 0.001, R^2^ = .54) and had higher MoRS values in cases vs controls (p < 0.001) (Figure 2A, middle plot). Our third and final model (i.e., MoRSAE), which used DNA methylation data adjusted for the two exposure variables as well as the other covariates in models 1 and 2, identified 4150 significant features that classified PTSD with 84% accuracy and an AUC of 89% (Figure 4, with precision, recall, and f1-score at 80%, 77%, and 78%, respectively (Table 2). As with models eMRS and MoRS, MoRSAE significantly predicted PTSD (beta = 1.20, p < 0.001, R^2^= 0.36) and had significantly (p < 0.001) different, and higher, MoRSAE in PTSD cases vs. controls (Figure 2A, right plot). In summary, while all three models produced risk scores that significantly predicted PTSD in the test dataset, and showed higher scores in aggregate between cases and controls, there was a decline in effect size (b) and explanatory power (R^2^) such that eMRS > MoRS > MoRSAE.

### Intercorrelation among study variables

A significant positive point-biserial correlation between eMRS and current PTSD was observed (ρ = .72, p < 0.001; Figure S3). Cumulative trauma (ρ = .40, p < 0.001) and childhood trauma (ρ = .57, p < 0.001) also showed a positive and significant correlation with eMRS. Notably, there was also a significant and positive point-biserial correlation (ρ = .62, p < 0.001) between methylation-only risk scores (MoRS) and PTSD, significant and positive correlation between cumulative trauma and MoRS (ρ = .16, p < 0.01) and childhood trauma and MoRS (ρ = .169, p < 0.01) (Figure S3). In contrast, while we observed a significant (p < 0.001) and positive point-biserial correlation (ρ = 0.49) between MoRSAE and PTSD (Figure S3), we observed a negative correlation between MoRSAE and cumulative trauma (ρ = −.13, p = 0.02) and childhood trauma (ρ = −.12, p = 0.03), respectively.

### Validation of Risk Scores in External Cohorts

We conducted external validation on risk scores from the three different models across four external cohorts— NCPTSD-TRACTS, BEAR, DCHS and PROGrESS. The NCPTSD-TRACTS cohort demonstrated a noticeable distinction (p < 0.05) in childhood trauma, but not in cumulative trauma (Table S1) between cases and controls. Similar to the discovery cohorts, the BEAR cohort exhibited a significant difference in both cumulative trauma and childhood trauma when comparing cases and controls. The DCHS cohort, on the other hand, only showed a significant difference in cumulative trauma, while the PROGrESS cohort did not display any significant difference in trauma variables between cases and controls.

The eMRS significantly predicted PTSD in one external cohort, BEAR (beta = 0.6839, p = 0.006) (Table S2); in this cohort, there was also a significant correlation (ρ = .24, p = 0.003) between eMRS and PTSD (Figure S4) and a significant difference in eMRS between PTSD cases and controls (p = 0.02, Figure S5). The eMRS did not significantly predict PTSD in any of the other three independent cohorts; however, the correlation between eMRS and PTSD showed the same (i.e., positive) direction of effect in the NCPTSD-TRACTS (beta = 0.0598, p = 0.35), PROGrESS (beta = 0.1141, p = 0.53) and DCHS (beta = 0.0631, p = 0.81) cohorts (Figures S6–S11). For model 2, the MoRS did not significantly predict PTSD in any external cohort (NCPTSD-TRACTS: beta = −0.0977, p = 0.28; BEAR: beta = 0.0239, p = 0.93; PROGrESS: beta = 0.2156, p = 0.52; DCHS: beta = 0.3739, p = 0.37). On the other hand, for model 3, the MoRSAE approached significance in association with PTSD in the NCPTSD-TRACTS cohort (beta = −0.1707, p = 0.05) and had significant difference in risk scores between cases and controls (p = 0.018) (Figure S7); however, the direction of effect was opposite to that observed in the discovery cohort.

### Testing of Pre-Deployment Risk scores to predict future PTSD 

**Figure 5:**
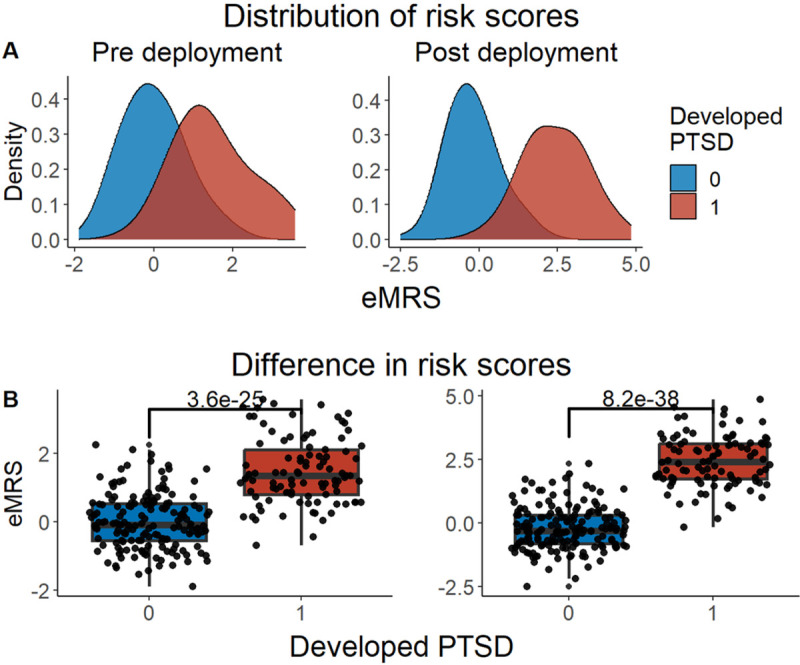
Distribution and difference in risk scores (eMRS) between PTSD cases and controls pre- and post-deployment (N = 262) — in figure legend, 0 is No PTSD and 1 is PTSD. A) The distribution of risk scores revealed that individuals who developed PTSD post-deployment had higher scores compared to those who did not, both before and after deployment. B) The difference in risk scores showed there was a significant (p < 0.001) difference in risk scores in those with PTSD post-deployment using Wilcox test.

**Figure 6:**
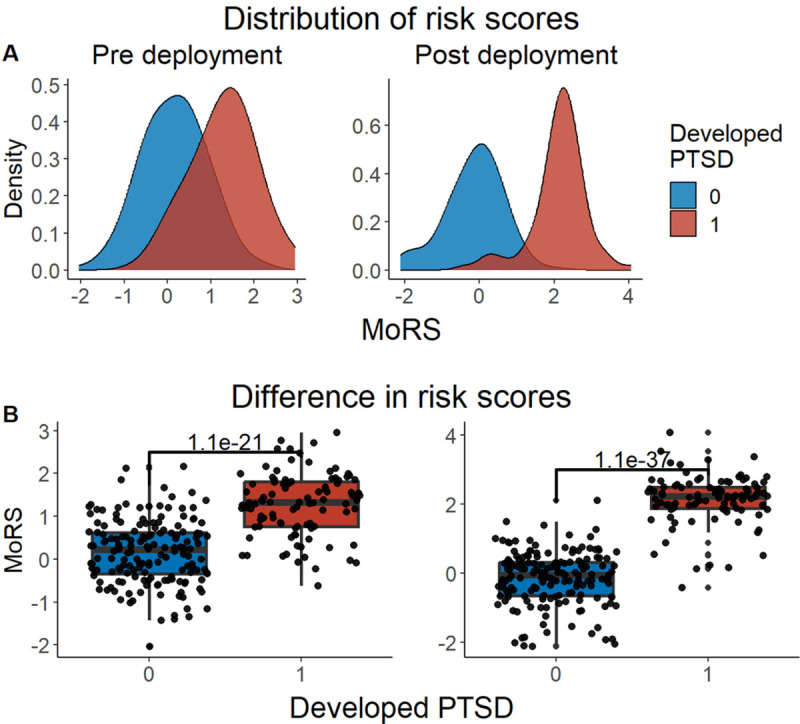
Distribution and difference in risk scores (MoRS) between cases and controls pre- and post-deployment (N = 262) — in figure legend, 0 is No PTSD and 1 is PTSD. A) Distribution of risk scores between cases and controls. Risk scores are higher in those who developed PTSD post-deployment than who didn’t in both pre and post deployment. B) Difference in risk scores between cases and controls. Wilcox test showed a significant difference (p < 0.001) in risk scores between cases and controls.

**Figure 7:**
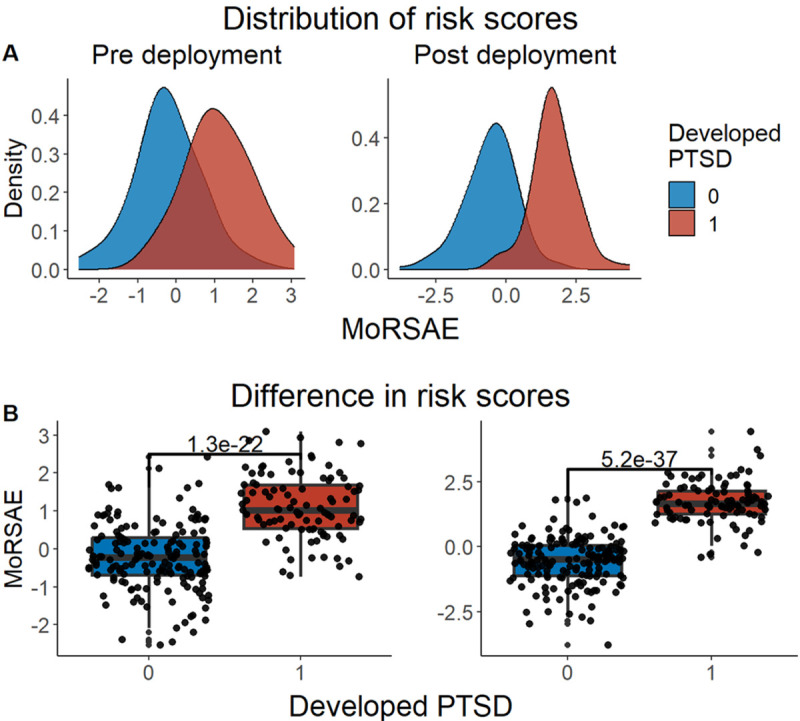
Distribution and difference in risk scores (MoRSAE) between cases and controls pre- and post-deployment (N = 262) — in figure legend, 0 is No PTSD and 1 is PTSD. A) The distribution of MoRSAE is higher in those who developed PTSD post-deployment B) The difference in risk scores showed there was a significant (p < 0.001) difference in risk scores in those with PTSD post-deployment using Wilcox test.

A compelling feature of risk scores is their ability to predict future disease risk. In our data, we were able to test the predictive ability of the MRS derived from our diagnostic/classification models on prospective risk of PTSD in two of our pre-deployment military cohorts, MRS I&II and Army STARRS (with data from the two cohorts analyzed together). MRS were calculated using “unseen” DNAm data from a pre-deployment timepoint, i.e. using DNAm data not included in the discovery cohort. All three models significantly predicted future PTSD based on risk scores calculated with pre-deployment data (eMRS beta = 1.92, p < 0.001, R^2^ = 0.53; MoRS beta = 1.99, p < 0.001, R^2^ = 0.46; and MoRSAE beta = 1.77, p < 0.001, R^2^ = 0.47) and had significant difference in risk scores between individuals who developed PTSD and those who did not (Figures 5, 6, 7).

### Assessment of Biological Significance among Important CpGs

Gene ontology (GO) analysis on the set of 3728 CpGs from models 1 and 2 revealed 403 nominally significant GO terms; among the 4150 important CpGs from Model 3, 382 nominally significant GO terms were identified. There were 115 GO terms common between models, including regulation of muscle adaptation, positive regulation of autophagy of mitochondrion, and sucrose metabolic process. Additionally, the Kyoto Encyclopedia of Genes and Genomes (KEGG) pathway analysis identified 47 pathways for models 1 and 2 and 25 pathways for model 3 at p < 0.05 (list of GO and KEGG in excel sheet as a supplementary file). Further, 14 pathways were common in models 1 and 2, and model 3, including, HIF-1 signaling pathway, mTOR signaling pathway, Insulin signaling pathway and Galactose metabolism. None of the GO terms or KEGG pathways passed the multiple hypothesis correction test.

## Discussion

It is crucial to identify individuals who are at a higher risk of developing PTSD in order to provide timely preventive measures and effective therapeutic interventions. Methylation risk scores (MRS) offer dynamic and modifiable genomic-based insights into disease risk. In this study, we leveraged machine learning and a diverse set of cohorts to develop MRS for PTSD, with an initial aim of distinguishing those with vs. without PTSD and to predict future PTSD cases. MRS derived from three different models demonstrated both high precision and high accuracy in predicting PTSD (i.e., identifying probable PTSD cases vs. controls) in the test dataset and, moreover, significantly predicted future PTSD. Although our approach did not yield MRS that consistently predict PTSD in independent cohorts fell short, our work leverages data from a diverse set of cohorts to develop what is, to our knowledge, the first methylation-based risk scores for PTSD. Future work that builds on these results will help to advance personalized preventive strategies and therapeutic interventions for PTSD in order to reduce the impact of this debilitating disorder on individuals and society.

Among the three models tested, the eMRS model showed the highest accuracy and precision to classify PTSD by using both exposure and DNA methylation variables. The inclusion of exposure variables substantially adds to the predictive power of the model. This finding aligns with the literature that suggests that experiencing trauma, particularly during childhood, significantly increases the likelihood of developing PTSD [47, [53, [54]. It is noteworthy that, despite not including any trauma exposure factors, the second model (MoRS) and third model (MoRSAE) that solely utilized methylation data in training still displayed notable predictive ability in the test dataset. These findings suggest that, even without using trauma variables in prediction, DNA methylation can still provide significant predictive information about PTSD. This also emphasizes the significant impact that trauma can have on the epigenetic landscape, which is consistent with other research studies [55, [56] that reported methylation differences linked to trauma. Overall, the decrease in classification accuracy across the models in the test dataset, from eMRS to MoRSAE, highlights the crucial role and predictive power that both methylation and increased trauma exposure have in forecasting PTSD. Also, the significant and strong correlation between eMRS, MoRS, MORSAE, and PTSD, respectively, indicates that these scores can aid in the early identification and risk assessment for individuals susceptible to PTSD and thus could be a valuable tool.

While validating our findings in the external cohorts, our findings were supported by the BEAR cohort for eMRS and NCPTSD-TRACTS cohort for MoRSAE, in terms of significant association and prediction for PTSD. This variability in validating the results in external cohorts could be due to individual differences in each cohort, for example differences in the type or severity of trauma in each cohort. For instance, similar to discovery cohorts, the BEAR cohort was the only cohort among the external cohorts that showed a significant difference in both cumulative and childhood trauma between the participants who had PTSD and those who didn’t. These differences emphasize the difficulty in creating predictive models and risk scores that are generalizable and that can apply to every cohort. Future work based on larger datasets may overcome this issue.

The ability to predict PTSD prior to deployment is particularly important, as deployment is linked to a higher probability of trauma exposure than typically observed in community samples and higher trauma load increases risk for PTSD [57]. It’s notable that all three models (eMRS, MoRS and MoRSAE) demonstrated significant capability in predicting future development of PTSD based on pre-deployment data, in particular since these data preceded trauma exposure and were not included in the training or testing phase of MRS model development. This type of predictive model and risk scores could be useful tool in predicting risk for future PTSD in populations with anticipated trauma exposure, and taking preventive measures to mitigate the risk.

Previous work has leveraged DNA methylation data as one among many biomarker types included in risk score approaches to predicting PTSD [58, [59]. An earlier study focused on war zone-related PTSD identified a set of 343 candidate biomarkers, of which 98 were DNA methylation values associated with particular genes [58]. From our identified list of significant CpGs (3728 in models 1 and 2), cg16335858 in GYLTL1B (*Glycosyltransferase-like 1B*) was previously identified as a biomarker in diagnosing war zone-related PTSD [58]. From the list of 4150 CpGs (model 3), one additional CpG, cg25448062 in *FADS1* (*Fatty acid desaturase 1*) was identified as a diagnostic biomarker in the same study. A subsequent study [59] showed prediction of post-deployment PTSD symptoms with the best AUC of 88% and CpGs cg01208318 and cg17137457 as top predictors but none of these were replicated in our study. More broadly, it is interesting to note that, 4 CpGs (cg04583842, cg04987734, cg16758086 and cg19719391) in genes *BANP, CDC42BPB, CHD5* and *Intergenic* respectively, have been associated with PTSD in recent PGC EWAS meta-analyses (Katrinli et al., submitted). Our results build on these earlier studies, highlighting novel CpGs that, when combined in a weighted, risk score format, may contribute to PTSD prediction.

While MRS identify CpGs as important features independent of their mechanistic contribution to the disease/phenotype in question, examining the functional significance associated with these important features may shed light on the biological processes implicated in the disease. In this study, gene ontology results provide interesting clues about the biological mechanisms that may be involved in the development of PTSD. For example, positive regulation of autophagy of mitochondrion, identified as nominally significant biological processes in all three models, is noteworthy, as prior research has suggested that autophagy plays a role in neurodegenerative illnesses [60, [61, [62], and exploring its connection to PTSD could provide insights into the disorder’s neurobiological underpinnings. Additionally, the link to sucrose metabolic process is intriguing and raises questions about the relationship between energy metabolism and stress responses [63], as metabolic disorders have been associated with PTSD [64]. Through our KEGG pathway analysis, we discovered additional implicated pathways, including mTOR and insulin signaling. These pathways play a crucial role in cellular growth and metabolism, highlighting the extensive physiological effects of PTSD beyond psychological distress [64, [65].

Our study is not without limitations. Chief among these is our external validation results: Even though we attempted to replicate the results from our models on external cohorts we found that some cohorts did not show significant correlations or associations. This indicates that there could be variability based on population characteristics, highlighting the importance of being cautious when generalizing our results. We note that, to date, attempts to validate risk scores in external, independent cohorts, as done in this study are not common, and most work focusses on reporting results based on validation in a test (i.e., internal) dataset [14]. Results from this work highlight the need to increase efforts to do so, in order to increase the generalizability of findings.

## Conclusions

We have presented three MRS that classify PTSD with high accuracy. Our models, especially a model that includes trauma exposure variables and DNAm (eMRS), reinforce earlier findings that methylation and trauma are interconnected and can be leveraged to increase the correct classification of those with vs. without PTSD, which may offer promising tools for early diagnosis and preventive strategies. Moreover, our models can potentially be a valuable tool in predicting the risk of developing PTSD in the future. Continued investigation into the functional significance of identified methylation features may help to shed additional light on the systemic pathology involved in this disorder. Finally, as more data become available, including additional molecular, environmental, and psychosocial factors in these scores may enhance their accuracy in predicting the condition and, relatedly, improve their performance in independent cohorts.

## Figures and Tables

**Table 1:** Demographic and clinical characteristics of the studies included in the discovery cohort.

	Current PTSD			
	Cases	Controls	*P value*	*Total*
**N**				
Army STARRS	42	111		153
DNHS	31	385		416
GTP	161	323		484
MRS I&II	63	60		123
PRISMO	17	33		50
All	314	912		1226
**Gender, Male (%)**				
Army STARRS	42 (27)	111 (73)		153 (100)
DNHS	10 (2)	161 (39)		171 (41)
GTP	25 (5)	107 (22)		132 (27)
MRS I&II	63 (51)	60 (49)		123 (100)
PRISMO	17 (34)	33 (66)		50 (100)
All	157 (50)	472 (51.8)		629 (51.3)
**Age, mean (SD)**				
Army STARRS	25.8 (5.1)	25.5 (5.2)	7.54E-01	25.6 (5.2)
DNHS	51.6 (11.1)	55.6 (17.1)	7.66E-02	55.3 (16.8)
GTP	41.7 (11.4)	42.4 (12.5)	5.48E-01	42.2 (12.1)
MRS I&II	23.3 (2.3)	22.9 (1.9)	3.59E-01	23.1 (2.1)
PRISMO	28.1 (10.1)	27.5 (9.1)	8.29E-01	27.7 (9.3)
All	36.1 (13.4)	44.1 (18)	5.89E-16	42.1 (17.3)
**PTSD symptoms severity, mean (SD)**				
Army STARRS	56.9 (9.6)	22.4 (5.8)	4.83E-28	32 (17)
DNHS	63 (16)	32.7 (11.4)	1.89E-11	34.9 (14.2)
GTP	70.4 (18.6)	25.1 (16.9)	2.17E-32	38.5 (27.1)
MRS I&II	65.4 (14.8)	13.6 (11.8)	1.30E-42	40.2 (29.2)
PRISMO	42 (4.4)	27 (4.8)	6.72E-13	32.1 (8.5)
All	63.1 (16.7)	27.7 (13.3)	5.88E-88	35.8 (20.5)
**Self-reported Race/Ethnicity, N (%)**				
Army STARRS				
African American	3 (2)	12 (7.8)		15 (9.8)
White	29 (19)	88 (57.5)	-	117 (76.5)
Other	10 (6.5)	11 (7.2)	-	21 (13.7)
DNHS			-	
African American	28 (6.7)	381 (91.6)	-	409 (98.3)
Other	3 (0.7)	4 (1)	-	7 (1.7)
GTP			-	
African American	153 (31.6)	307 (63.4)	-	460 (95)
Other	8 (1.7)	16 (3.3)	-	24 (5)
MRS I&II			-	
African American	2 (1.6)	2 (1.6)		4 (3.3)
White	53 (43.1)	53 (43.1)	-	106 (86.2)
Other	8 (6.5)	5 (4.1)	-	13 (10.6)
PRISMO			_-_	
African American	1 (2)	1 (2)	-	2 (4)
White	11 (22)	27 (54)	-	38 (76)
Other	5 (10)	5 (10)	-	10 (20)
All				
African American	187 (59.6)	703 (77.1)	-	890 (72.6)
White	93 (29.6)	168 (18.4)	-	261 (21.3)
Other	34 (10.8)	41 (4.5)	-	75 (6.1)
**Smoking Score, mean (SD)**				
Army STARRS	−5.4 (18.4)	−7.8 (18)	4.75E-01	−7.1 (18.1)
DNHS	3.8 (30.5)	−0.6 (33)	4.45E-01	−0.3 (32.8)
GTP	−4.1 (35.4)	−2.8 (35.4)	7.05E-01	−3.3 (35.4)
MRS I&II	−8.5 (17)	−10.8 (15)	4.43E-01	−9.6 (16)
PRISMO	1.3 (16.9)	2 (21.3)	9.06E-01	1.7 (19.7)
All	−4.1 (29.3)	−2.9 (31.3)	5.21E-01	−3.2 (30.8)
**Childhood Trauma, mean (SD)**				
Army STARRS	7.1 (3.3)	6.3 (2.2)	1.44E-01	6.5 (2.5)
DNHS	7.6 (5.7)	4.4 (3.4)	4.44E-03	4.7 (3.7)
GTP	56.1 (20.1)	37.7 (13.4)	1.67E-21	43.8 (18.1)
MRS I&II	41.7 (12.2)	37.5 (10.4)	4.10E-02	39.6 (11.5)
PRISMO	5.5 (2.6)	2.8 (2.2)	1.03E-03	3.7 (2.7)
All	39.1 (26.2)	18.5 (18.5)	3.3E-32	23.8 (22.6)
**Cumulative Trauma, Mean (SD)**				
Army STARRS	1 (0)	1 (0)	-	1 (0)
DNHS	12.2 (7)	6.1 (4.1)	3.63E-05	6.6 (4.7)
GTP	7 (3.1)	4.4 (2.8)	2.33E-17	5.3 (3.1)
MRS I&II	11.2 (2.9)	10.3 (3.8)	0.125	10.8 (3.4)
PRISMO	6.5 (3.1)	5.9 (3.8)	0.557	6.1 (3.5)
All	7.6 (4.8)	5.2 (4)	9.45E-15	5.8 (4.3)

**Table 2:** Model performance in accuracy, precision, recall, f1-score, and AUC. Elastic net performed best with an accuracy of 92%.

Model	Accuracy (%)	Precision (%)	Recall (%)	F1-score (%)	AUC (%)
**Elastic Net (Model 1)**	92	91	87	89	96
**Elastic Net (Model 2)**	89	86	83	84	95
**Elastic Net (Model 3)**	84	80	77	78	89

## Data Availability

Owing to military cohort data sharing restrictions, data from MRS I& II, Army STARRS, PRISMO, and NCPTSD-TRACTS cannot be publicly posted. For other cohorts, individual-level data from the cohorts or cohort-level summary statistics may be made available to researchers following an approved analysis proposal through the PGC Post-traumatic Stress Disorder EWAS group with agreement of the cohort PIs. For additional information on access to these data, including PI contact information for the contributing cohorts, please contact the corresponding author.
